# Cohort Profile: The Finnish Gestational Diabetes (FinnGeDi) Study

**DOI:** 10.1093/ije/dyaa039

**Published:** 2020-05-06

**Authors:** Elina Keikkala, Sanna Mustaniemi, Sanna Koivunen, Jenni Kinnunen, Matti Viljakainen, Tuija Männisto, Hilkka Ijäs, Anneli Pouta, Risto Kaaja, Johan G Eriksson, Hannele Laivuori, Mika Gissler, Tiina-Liisa Erkinheimo, Ritva Keravuo, Merja Huttunen, Jenni Metsälä, Beata Stach-Lempinen, Miira M Klemetti, Minna Tikkanen, Eero Kajantie, Marja Vääräsmäki

**Affiliations:** d1 PEDEGO Research Unit, Medical Research Centre Oulu, Oulu University Hospital and University of Oulu, Oulu, Finland; d2 Public Health Promotion Unit, National Institute for Health and Welfare, Helsinki and Oulu, Finland; d3 Northern Finland Laboratory Centre NordLab, Department of Clinical Chemistry and MRC Oulu, Oulu University Hospital and the University of Oulu, Oulu, Finland; d4 Department of Government Services, National Institute for Health and Welfare, Helsinki, Finland; d5 University of Turku and Turku University Hospital, Institute of Clinical Medicine, Internal Medicine, Turku, Finland; d6 Department of General Practice and Primary Health Care, University of Helsinki and Helsinki University Hospital, Helsinki, Finland; d7 Folkhälsan Research Center, Helsinki, Finland; d8 Singapore Institute for Clinical Sciences, Agency for Science, Technology, and Research, Singapore; d9 Department of Obstetrics and Gynaecology, Yong Loo Lin School of Medicine, National University of Singapore, Singapore; d10 Department of Obstetrics and Gynaecology, Tampere University Hospital and Tampere University, Faculty of Medicine and Health Technology, Tampere, Finland; d11 Medical and Clinical Genetics, University of Helsinki and Helsinki University Hospital, Helsinki, Finland; d12 Institute for Molecular Medicine Finland, Helsinki Institute of Life Science, University of Helsinki, Helsinki, Finland; d13 National Institute for Health and Welfare, Information Services Department, Helsinki, Finland; d14 Karolinska Institute, Department of Neurobiology, Care Sciences and Society, Stockholm, Sweden; d15 Department of Obstetrics and Gynaecology, Hospital District of South Ostrobothnia, Seinäjoki, Finland; d16 Department of Obstetrics and Gynaecology, Kainuu Central Hospital, Kajaani, Finland; d17 Department of Obstetrics and Gynaecology, Satakunta Health Care District, Pori, Finland; d18 Department of Obstetrics and Gynaecology, Central Finland Health Care District, Jyväskylä, Finland; d19 Department of Obstetrics and Gynaecology, South Karelia Social and Health Care District, Lappeenranta, Finland; d20 Department of Obstetrics and Gynaecology, University of Helsinki and Helsinki University Hospital, Helsinki, Finland; d21 Lunenfeld-Tanenbaum Research Institute, Mount Sinai Hospital, Toronto, ON, Canada; d22 Children’s Hospital, University of Helsinki and Helsinki University Hospital, Helsinki, Finland; d23 Department of Clinical and Molecular Medicine, Norwegian University of Science and Technology, Trondheim, Norway

## Why was the cohort set up?

The Finnish Gestational Diabetes (FinnGeDi) study is a multicentre study that considered Finnish women who gave birth in 2009–12, as well as their children and the children’s fathers. The study period was after the introduction of new Finnish national comprehensive screening guidelines for gestational diabetes mellitus (GDM).^1^ The study consisted of two arms: a prospective clinical, genetic case-control arm and a national register-based arm which also includes data on children’s siblings and grandparents. The FinnGeDi study was initiated to study different aspects of GDM as diagnosed by comprehensive screening, which was expected to increase the prevalence of GDM by identifying previously undiagnosed cases.^2^

GDM is characterized by carbohydrate intolerance and/or hyperglycaemia—with its onset or first recognition during pregnancy, which is not overt type 1 diabetes nor type 2 diabetes (T2D).^3^ GDM affects 10–30% of all pregnancies,^4^ recurs in 30–84% of women^5^ and is becoming more common worldwide.^6^ It is frequently the first manifestation of an increased risk of diabetes, as up to two-thirds of women with a history of GDM are estimated to develop subsequent T2D.^7–9^ Women with a history of GDM also have an increased risk for other metabolic and cardiovascular diseases.^9^^,^^10^ Exposure to maternal hyperglycaemia also impacts on the fetus: in addition to short-term consequences—that is, macrosomia and neonatal hypoglycaemia^11^—children born from GDM pregnancies are at increased risk of later T2D, metabolic syndrome, cardiovascular disease and cognitive impairment.^12–14^

GDM represents a part of a continuum of maternal hyperglycaemia.^2^^,^^11^ There are no unanimously accepted international criteria for diagnosis or screening,^15^ and guidelines vary considerably even between high-income countries.^15–17^ Typically, GDM is diagnosed by an oral glucose tolerance test (OGTT), which may be performed only in women whose characteristics indicate an increased risk (risk-factor-based screening) or in all or most pregnant women (universal or comprehensive screening).^15^ The FinnGeDi study was established after the national Finnish Current Cure Guidelines were introduced in 2008 and comprehensive screening was recommended to replace the previous risk-factor-based screening.^1^ The study was expected to identify new GDM cases in women without previous risk factors and result in a higher GDM prevalence.^2^

The study aimed to identify potential genetic and epigenetic biomarkers of GDM and assess putative risk factors and clinical characteristics of GDM, enabling the characterization of clinically identifiable and mechanistically meaningful subgroups of the disorder. The short- and long-term health of the mother and child are to be followed up—that is, evaluating the consequences of GDM. Furthermore, the incidence, distribution and consequences of GDM are to be assessed in different socioeconomic and demographic groups and across generations. To approach these questions from different perspectives, two arms were included in the FinnGeDi study: (i) a multicentre case-control arm including questionnaires, medical data, Medical Birth Register (MBR) data and DNA samples from pregnant women with and without GDM, their children and the children’s fathers; and (ii) the register-based arm using the MBR and other Finnish comprehensive national registers. The study headquarters and database are located at the National Institute for Health and Welfare (Finland), which is the primary research institution of the study in addition to Oulu University Hospital. The study is funded by the Academy of Finland and private foundations.

## Who is in the cohort?

The cohort includes two arms: a case-control arm and a register-based arm.

### Case-control arm

**Figure 1:**
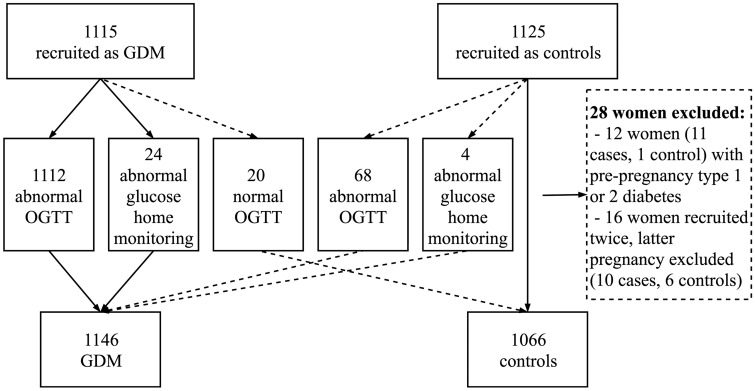
Flow chart of women in the case-control arm. GDM, gestational diabetes mellitus; OGTT, oral glucose tolerance test.

The prospectively collected case-control cohort consists of 1146 pregnant women with GDM and 1066 women without GDM, their children from the index pregnancy and the children’s fathers. The flow chart of the study population is presented in Figure 1. Women with GDM were recruited from delivery units as they came to give birth, and the next consenting woman without GDM was recruited as a control. The women were recruited between 1 February 2009 and 31 December 2012 at two tertiary-level hospitals (Oulu University Hospital and Helsinki University Hospital), which serve as secondary-level hospitals for their region, and five secondary-level hospitals (in Jyväskylä, Pori, Kajaani, Seinäjoki and Lappeenranta). All the hospitals serve a specific geographical area. Women with pre-pregnancy diabetes mellitus (DM) and multiple pregnancies were excluded from the study. Women and their spouses (the fathers of the children) signed informed consent to the use of the growth and developmental data of their children and to contact with the family later for follow-up studies. Blood samples (leukocyte DNA) were drawn from both parents and from the umbilical cord after delivery. Plasma from the umbilical cord sample was frozen and stored at –80°C. The parents completed background questionnaires—including information on family and medical history and lifestyle factors (i.e. physical activity, diet and smoking). Maternal welfare clinical and hospital records were reviewed to confirm GDM diagnosis, and detailed information on the women’s medical and obstetric history, pregnancy complications and outcomes, laboratory measurements and the newborns’ health was obtained. These data were combined with the MBR data. For each delivery in Finland, a structured form for the MBR is completed by the health personnel at the delivery hospital within 7 days after delivery. It included data on key obstetric, perinatal and neonatal outcomes. The MBR was completed using data compiled by the Population Register Centre on live births and by Statistics Finland on stillbirths and infant deaths. Available data, including blood samples, are described in detail in Tables 1 and 2.

**Table 1.** Number of available samples and data in the case-control arm

Sample/data	GDM *n* = 1146			Control *n* =1066		
	Mother *n* (%)	Father *n* (%)	Child *n* (%)	Mother *n* (%)	Father *n* (%)	Child *n* (%)
DNA	1044 (91.1)	910 (79.4)	1046 (91.3)	1013 (95.0)	893 (83.8)	957 (89.8)
Cord plasma			1051 (91.7)			967 (90.7)
Questionnaire	1030 (89.9)	599 (50.5)		935 (87.7)	586 (49.5)	
Medical records	1117 (97.5)		1117 (97.5)	1042 (97.7)		1042 (97.7)
Medical Birth Register	1146 (100)			1066 (100)		

DNA duo: DNA samples from mother and child; GDM *n* = 971 (84.7%)/control *n* = 927 (87.0%).

DNA trio: DNA samples from mother, father and child; GDM *n* = 846 (73.8%)/control *n* = 833 (78.1%).

GDM, gestational diabetes mellitus.

**Table 2.** Description of the data sources for both study arms

Register/source	Type	Data	Time	Subject	Arm
Medical records	Hospital and primary health care records	Index pregnancy and delivery data OGTT values	Baseline	Mo	Case-control
		Delivery data Primary health care data (growth, development, health)	Baseline Follow-up	C	Case-control
Questionnaire		Background characteristics, lifestyle factors, family history	Baseline	Mo/Fa	Case-control
National Institute for Health and Welfare	Medical Birth Register	Identification of the index women and pregnancy data	Baseline	Mo	Case-control Register-based
		Previous and following pregnancies	Baseline Follow-up	Mo	Case-control Register-based
		Births of the parents	Baseline	Mo/Fa	Register-based
	Register on congenital malformations		Baseline Follow-up	Mo/Fa/C Mo/Fa/C/S	Case-control Register-based
	Care Register for Health Care (HILMO)	Diagnoses Procedures Hospitalization	Baseline Follow-up	Mo/Fa/C Mo/Fa/C/S/G	Case-control Register-based
	Register of Primary Health Care Visits (AvoHILMO)	Reasons for visits/diagnosesProceduresOutpatient visits	Follow-up (from 2011^a^)	Mo/Fa/C Mo/Fa/C/S/G	Case-control Register-based
	Register of Social Welfare Benefits		Years 2005-09	Mo/Fa	Register-based
	Cancer Register		Baseline Follow-up	Mo/Fa/C Mo/Fa/C/G	Case-control Register-based
	Cancer Screening Registry	Breast cancer screening	Follow-up	Mo	Case-control Register-based
		Cervical cancer screening		Mo	Case-control Register-based
Statistics Finland	Educational degree and occupation		Baseline	Mo/Fa	Register-based
	Income and socioeconomic status		Years 2005–09	Mo/Fa	Register-based
	Date and causes of death		Follow-up	Mo/Fa/C Mo/Fa/C/S/G	Case-control Register-based
Population Register Centre	Identification of the father and grandparents of index children		Baseline	Fa/G	Register-based
	Identification of previous children		Baseline	Fa	Register-based
Social Insurance Institution of Finland		Reimbursement of drugs	Baseline Follow-up	Mo/Fa/C Mo/Fa/C/S/G	Case-control Register-based
		Purchase of medicine	Follow-up	Mo/C	Case-control Register-based
	Prescription centre and archive	Electronic prescriptions	Follow-up (from 2017^a^)	Mo/C	Case-control Register-based
Matriculation Examination Board	Matriculation examination scores			Mo/Fa	Register-based
DNA sample data		Epigenetic and genetic data	Baseline	Mo/Fa/C	Case-control
Biobank Borealis	Finnish Maternity Cohort Biobank	Maternal first trimester serum sample	Baseline	Mo	Case-control

Mo, index mother; Fa, index father; C, index child; S, siblings of the index child; G, grandparents of the index child; OGTT, oral glucose tolerance test

a Year when register was established.

The diagnosis of GDM was based on an abnormal OGTT result during pregnancy. According to the Finnish Current Care guidelines introduced in 2008, a 75 g 2-h OGTT was recommended to be performed between the 24th and 28th gestational weeks in all women except those with a very low risk of developing GDM. For high-risk women, OGTT was recommended between 12 and 16 weeks of pregnancy, and if normal, a repeat test was recommended between 24 and 28 weeks. The detailed screening criteria are described in Table 3. The cut-off concentrations for venous plasma glucose were ≥5.3 mmol/l at baseline (fasting glucose), ≥10.0 mmol/l at 1 h after glucose intake or ≥8.6 mmol/l at 2 h after glucose intake. GDM diagnosis was set if one or more glucose concentrations exceeded the cut-off levels.^1^

**Table 3.** Current Care Guideline 2007 for the screening of gestational diabetes mellitus using oral glucose tolerance test in Finland (Current Care Guideline: Gestational diabetes 2007)^1^

Screening	Pregnancy weeks	Criteria
OGTT	12–16	Previous GDM diagnosis Prepregnancy BMI ≥35 kg/m^2^ Glucosuria in early pregnancy Oral glucocorticoid medication Family history of T2D (parents, grandparents, siblings and children) Polycystic ovary sydrome
OGTT	24–28	Recommended to be performed for all pregnant women (exceptions detailed above)
No OGTT		Primiparous: age <25 years, pre-pregnancy BMI<25 kg/m^2^ and no family history of T2D Multiparous: age <40 years, pre-pregnancy BMI <25 kg/m^2^ and no previous GDM diagnosis or macrosomia

OGTT, oral glucose tolerance test; GDM, gestational diabetes mellitus; BMI, body mass index; T2D, type 2 diabetes mellitus.

Comparisons between women with or without GDM and their spouses are shown in Table 4. As expected, women with GDM were older, more often multiparous, had higher prepregnancy body mass index (BMI) values and often had chronic hypertension compared with controls. Less upper tertiary-level educated women were in the GDM group than in the control group. The groups were comparable in terms of smoking before and during pregnancy. The incidence of gestational hypertension and preeclampsia was higher in the women with GDM than in the controls. For preeclampsia, the difference remained significant even after adjustment for parity, maternal age and pre-pregnancy BMI. Women with GDM had more inductions of labour, caesarean sections and large-for-gestational-age (LGA) newborns than controls. The spouses of women with GDM were older and had higher BMI than those of the control group. The screening rates and glucose metabolism status of women with or without GDM are given in Supplementary Table 1, available as Supplementary data at *IJE* online.

**Table 4.** Maternal, neonatal and paternal characteristics of participants in the case-control arm

	GDM *n* =1146	Control *n*=1066	***P*-value** ^a^	***P*-value** ^b^
Maternal characteristics				
Age at delivery, years	32.1 ± 5.4	29.6 ± 5.2	<0.001	
Gravity, *n*	1.9 ± 2.5	1.6 ± 2.2	<0.001	
Parity, *n*	1.3 ± 2.0	1.1 ± 1.8	0.014	
Primiparous*, n* (%)	482 (42.1%)	520 (48.8%)	0.002	
Weight, kg (self-reported, pre-pregnancy)	76.6 ± 17.2 (1145)	64.8 ± 12.4	<0.001	<0.001^c^
Height, m (self-reported)	164.8 ± 5.8	165.5 ± 5.9	0.005	
BMI, kg/m^2^ (self-reported, pre-pregnancy)	28.2 ± 6.1 (1145)	23.6 ± 4.2	<0.001	<0.001^c^
Education % (self-reported)	(1030)	(935)	0.014	
Basic or less, *n*	68 (6.6%)	42 (4.5%)		
Secondary, *n*	486 (47.2%)	426 (45.6%)		
Lower-level tertiary, *n*	270 (26.2%)	231 (24.7%)		
Upper-level tertiary, *n*	206 (20.0%)	236 (25.2%)		
Smoking before pregnancy, *n* (%)	340 (31.1%) (1094)	298 (30.1%) (990)	0.629	
Smoking during pregnancy, *n* (%)	191 (16.7%) (1142)	161 (15.1%) (1065)	0.303	
Gestational weight gain, kg^d^	12.3 ± 5.8 (1055)	14.8 ± 5.1 (1032)	<0.001	<0.001^c^
Excess gestational weight gain^e^, *n* (%)	521 (49.4%)	470 (45.5%)	0.079	0.006^c^
Chronic hypertension, *n* (%)^f^	181 (15.8%) (1144)	54 (5.1%)	<0.001	0.011^g^
Gestational hypertension, *n* (%)^h^	235 (20.5%) (1144)	151 (14.2%)	<0.001	0.134^g^
Preeclampsia, *n* (%)^i^	70 (6.1%) (1144)	28 (2.6%)	<0.001	0.016^g^
Induced labour, *n* (%)	515 (44.9%)	342 (32.1%)	<0.001	0.012^g^
Gestational weeks at delivery	39.6 ± 1.4	40.1 ± 1.4	<0.001	<0.001^g^
<37 weeks, *n* (%)	41 (3.6%)	23 (2.2%)	0.046	0.302^j^
≥42 weeks, *n* (%)	16 (1.4%)	30 (2.8%)	0.020	0.012^j^
Mode of delivery, *n* (%)				
Vaginal, *n* (%)	912 (79.6%)	923 (86.6%)	<0.001	
Vacuum extraction, *n* (%)	109 (9.5%)	129 (12.1%)	0.050	0.228
Caesarean section	234 (20.4%)	143 (13.4%)	<0.001	
Neonatal characteristics				
Five-minute Apgar points <7, *n* (%)	26 (2.6%) (999)	20 (2.1%) (937)	0.499	
Shoulder dystocia, *n* (%)	5 (0.4%)	4 (0.4%)	0.822	
Erb’s palsy, *n* (%)	1 (0.1%)	(0.0%)	0.355	
Birthweight, g	3647 ± 507	3570 ± 496	<0.001	<0.001^k^
Relative birthweight, SD	0.2 ± 1.1	−0.1 ± 1.0	<0.001	<0.001^k^
Birthweight ≥ 4500 g, *n* (%)	33 (2.9%)	24 (2.3%)	0.351	
LGA, *n* (%)	64 (5.6%)	28 (2.6%)	<0.001	0.214^g^
SGA, *n* (%)	21 (1.8%)	34 (3.2%)	0.041	0.240^g^
Paternal characteristics				
Age, years	33.9 ± 6.2 (984)	31.5 ± 5.7 (933)	<0.001	
BMI, kg/m^2^ (self-reported)	27.0 ± 3.9 (591)	26.2 ± 3.7 (578)	<0.001	

Data are presented as mean ± SD or as number (percentages).

GDM, gestational diabetes mellitus; BMI, body mass index; LGA, large for gestational age (birthweight ≥ 2 SD); SGA, small for gestational age (birthweight ≤2 SD).

a Unadjusted *P*-values based on Student’s t test or χ^2^ test.

b Adjusted *P*-values based on logistic regression.

c Adjusted for parity and mother’s age at birth.

d Difference of (self-reported) pre-pregnancy weight and weight at the last antenatal visit at 35 gestational weeks or later.

e Excess gestational weight gain based on Institute of Medicine 2009 criteria.

f Systolic blood pressure ≥ 140 mmHg and/or diastolic blood pressure ≥ 90 mmHg detected before 20 weeks of gestation.

g Adjusted for parity, mother’s age at birth and pre-pregnancy BMI.

h Blood pressure ≥ 140/90 mmHg, no proteinuria.

i Blood pressure ≥ 140/90 mmHg and proteinuria (≥ 0.3 g/24 h or two ≥ 1+ readings on a dipstick).

j Adjusted for parity, mother’s age at birth, pre-pregnancy BMI, hypertensive pregnancy complications and induction of labour (yes/no).

k Adjusted for parity, mother’s age at birth, gestational weeks, pre-pregnancy BMI and hypertensive pregnancy complications.

### Register-based arm

The register-based arm includes all 59 057 singleton pregnancies in women who gave birth in Finland in 2009. They were identified through the MBR, which includes data on whether OGTT was ‘performed (yes/no)’ and ‘abnormal OGTTs (yes)’, if ‘insulin treatment was begun during pregnancy (yes)’ and ‘ICD-10 diagnosis codes of GDM’. The accuracy of different variables and their combinations to identify GDM cases was checked against laboratory-verified OGTT results. In addition, data from the Finnish Care Register for Health Care (HILMO, former Hospital Discharge Register) were tested to identify whether it improved the accuracy of MBR variables (Supplementary Data 1, available as Supplementary data at *IJE* online). Based on these results, the accuracy of all three MBR variables mentioned above without HILMO variables was found to be 94.3%, and they were used to identify GDM cases from register data.

**Figure 2:**
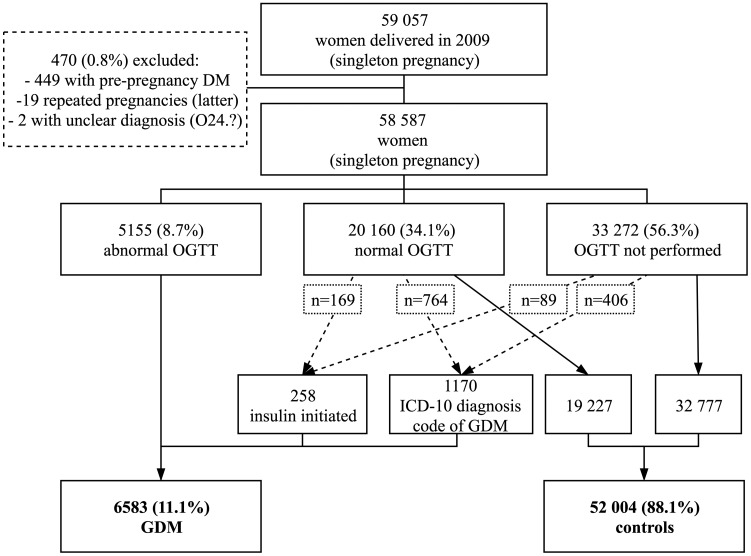
Flow chart of women in the register-based arm according to the Medical Birth Register 2009. Number of women (% of all 59 057 singleton pregnancies). DM, diabetes; OGTT, oral glucose tolerance test; GDM, gestational diabetes mellitus.

In 2009, a total of 6583 women (11.1%) were reported to have GDM according to an ‘abnormal OGTT finding’ and/or ‘insulin initiation during pregnancy’ and/or ‘ICD-10 diagnosis codes of GDM’ (ICD-10 code ‘O24.4’ or ‘O24.9’). Women with type 1 diabetes and T2D (*n* = 449), women with unclear diagnosis codes (*n* = 2) and the latter pregnancy of women with two pregnancies in 2009 (*n*=19) were excluded. All other women were chosen to serve as controls (*n* =52 004) (Figure 2). Comparison of the baseline clinical characteristics of women with GDM and controls is shown in Supplementary Table 2A, available as Supplementary data at *IJE* online. OGTT-verified controls (*n*=19 227) were found to have more background risk factors of GDM than controls without OGTT results (*n*=32 777) (Supplementary Table 2B, available as Supplementary data at *IJE* online). Women recognized as having GDM through the MBR variable ‘ICD-10 diagnosis code of GDM’ had higher parity than women who were recorded to have ‘abnormal OGTT’ and/or ‘insulin initiation during pregnancy’ in the MBR (Supplementary Table 2C, available as Supplementary data at *IJE* online).

The children born in 2009 serve as index children for the identification of their siblings, fathers and grandparents. By using the unique personal identification code allocated to each citizen and permanent resident of Finland, data from various national registers (including data on, for example, hospital discharges and diagnoses, reimbursement for drugs, congenital anomalies, cancer diagnoses, time and causes of deaths, social welfare benefits, educational degrees and occupation and matriculation examination scores) can be linked to all family members (Table 2). According to Finnish legislation, a register study does not require permission from the study participants if they are not contacted due to the study.

As the MBR does not include numerical OGTT data, these data were obtained from hospital laboratory databases for a subpopulation of 4954 women with singleton pregnancies, who delivered in 2009 in six out of seven study hospitals, with a total of 15 000 births per year. These data were also used to validate the register data (Supplementary Figure 1, available as Supplementary data at *IJE* online).

## How often have they been followed up?

In the case-control arm, the questionnaires, medical data from hospital records and baseline register data were collected at the time of enrolment in 2009–12. The study enables longitudinal follow-up for both women and children by combining these data with data obtained from national registers. The development and growth data of the children will be collected later from child welfare clinic records. In the register-based arm, the register data from MBR and the OGTT results of the subpopulation of 4954 women were collected at baseline in 2009. The first follow-up for the both arms will be performed 7–10 years after the completion of the enrolment, and is planned to continue for decades. Permissions for the register follow-ups will be updated in 2024 and after that in 5-year periods. The linkage to registers is presented in Table 2.

## What has been measured?

The case-control cohort provides a large dataset from questionnaires, hospital records and national registers, combined with DNA trio samples from parents and children to study novel genetic and epigenetic markers of GDM (Tables 1 and 2)

The register-based arm provides data from MBR and other national registers including registers maintained by the National Institute of Health and Welfare, Statistics Finland, Population Register Centre and Social Insurance Institution of Finland (Table 2). Index mothers and their children are identified from MBR records, and the fathers, siblings and grandparents of the index children are identified from the Population Register Centre. The linkage of these registers provides extensive data on diseases and medical conditions with their complications and socioeconomic adversities of the index families.

## What has been found? Key findings and publications

In the case-control arm, blood samples and data to study epigenetics of GDM have been collected and discovery analyses have been performed. The study will proceed to epigenetic replication in other collaborative cohorts. The results have not yet been published. In multivariate analyses of clinical data, women’s own preterm birth, pre-pregnancy obesity, age ≥35 years and family history of GDM or T2D were found to be independent risk factors for GDM.^18^ In the register-based arm, an article focusing on OGTT results after 24 weeks of pregnancy in the subpopulation of 4033 women has been published.^19^

## What are the main strengths and weaknesses?

The main strengths of the population-based FinnGeDi cohort include prospective case-control samples from women, children and their fathers to study genetics and epigenetics of GDM; and the large and comprehensive databases of clinical, lifestyle and register data of women and children, with possibilities of longitudinal follow-up. The use of different registers enables a multifaceted assessment of the underlying socioeconomic and educational background which may affect the prevalence and consequences of GDM. The extension of data collection to the children’s grandparents will contribute to the assessment of intergenerational effects on GDM.

In the case-control arm, OGTT was performed in 672 of the 1066 women (62.8%) in the control group. A total of 319 (81%) of those 394 women without OGTT did not enter the screening because they were estimated to be at very low risk of developing GDM according to the national guidelines.^1^ Clinical characteristics of the women without OGTT are detailed in Supplementary Table 3, available as Supplementary data at *IJE* online.

In the register-based arm, GDM status is based on register data, the validity of which to identify GDM has been evaluated as high (Supplementary Data 1, available as Supplementary data at *IJE* online). In general, the quality of Finnish national registers, especially MBR, is high and the coverage complete.^20^^,^^21^ In the control group, only one-third of women were verified to have normal OGTT results (Figure 2). However, controls without OGTT results were found to have less GDM risk factors than controls having normal OGTT results (Supplementary Table 2B, available as Supplementary data at *IJE* online).

The use of comprehensive screening has resulted in an increase in the incidence of GDM during recent years.^22^^,^^23^ The screening frequency has increased from 51.4% in 2009–12 to 66.0% in 2018, and the prevalence of GDM increased from 11.3% to 21.3%, respectively.^24^ Thus, some women with GDM remained undiagnosed when our study was conducted.

## Can I get hold of the data? Where can I find out more?

Access to clinical data is regulated by ethics approvals and individual consent. Access to registry data is subject to permission from the registry authorities. For enquiries regarding possible collaboration, please contact FinnGeDi’s principal investigator and study coordinator, Adjunct Professor Marja Vääräsmäki, MD, PhD: [marja.vaarasmaki@oulu.fi] or Marja Vääräsmäki, Oulu University Hospital, Department of Obstetrics and Gynaecology, PO Box 23, 90029 OYS, Oulu, Finland.

**Profile in a nutshell**

- The FinnGeDi cohort was set up to provide a database combining detailed clinical data and DNA trio samples from mother, father and child to study genetics, epigenetics, phenotype and long-term consequences of GDM diagnosed using the new comprehensive screening guidelines.
- The cohort is based at the National Institute for Health and Welfare (Oulu, Finland).
  - The case-control cohort was recruited in 2009–12 and includes 1146 women with GDM and 1066 non-diabetic controls aged 17–48 years, their children and the children’s fathers.
  - The register-based cohort consists of Finnish families where a mother gave birth in 2009 (*n* = 59 057 singleton pregnancies). This cohort includes 6583 women (11.1%) with GDM.
- The main categories of data were blood samples from parents and children, clinical data from hospital and maternal welfare clinic records, register data from national registers and self-reported lifestyle and medical and family history data from questionnaires.
- Follow-up data collection will be performed 7–10 years after the end of the recruitment for both cohorts, and is planned to continue for decades. This will include the linkage of baseline data to national registers—for example, hospital discharge diagnoses, data on reimbursement for and purchase of drugs and time and causes of deaths. Registers are updated annually.
- The data cannot be provided as open access due to strict national data protection regulations, but we welcome collaboration. The use of registry data requires study permission from all national registry authorities. Requests may be addressed to [marja.vaarasmaki@oulu.fi].

## Supplementary Data

Supplementary data are available at *IJE* online.

## Funding

The study is funded by Academy of Finland, Diabetes Research Foundation, Foundation for Pediatric Research, Juho Vainio Foundation, Novo Nordisk Foundation, Signe and Ane Gyllenberg Foundation, Sigrid Jusélius Foundation, Yrjö Jahnsson Foundation, Finnish Medical Foundation, Research Funds of Oulu University Hospital (state grants), Research Funds of Helsinki University Hospital (state grants), Medical Research Center Oulu and National Institute for Health and Welfare (Finland).

## Supplementary Material

dyaa039_Supplementary_Data
