# Supplementary material for: Cohort Profile: The Finnish Gestational Diabetes (FinnGeDi) Study
Source: Int J Epidemiol. 2020 May 6;49(3):762–763g. doi: 10.1093/ije/dyaa039 (PMC7394962; doi:10.1093/ije/dyaa039)
Supplement: dyaa039_Supplementary_Data [file dyaa039_supplementary_data.zip › dyaa039-Suppl_Data/Supplement Data 1. Accuracy of register variables.docx]

**Supplement Data 1. Accuracy of register variables.**

**Objective**

The data validation using a subcohort of women with detailed oral glucose tolerance test (OGTT) laboratory results were used to 1) evaluate the accuracy of different variables of the Medical Birth Register (MBR) to identify gestational diabetes mellitus (GDM) cases and 2) evaluate whether the accuracy is improved when adding variables from the Care Register for Health Care (HILMO, former Hospital Discharge Register) to MBR variables. The HILMO data included main and additional ICD-10 diagnosis codes set during all in- and outpatient visits in Finnish hospitals since 1998.

**Methods**

Data on 75 g 2-hour oral glucose tolerance test (OGTT) were collected from the databases of six central laboratories serving two tertiary- and four secondary-level delivery hospitals (in Oulu, Tampere, Kajaani, Pori, Lappeenranta and Seinäjoki) between April 2008 and December 2009. The data included date and time of OGTT and plasma glucose concentrations at 0 h (fasting glucose), 1 h and 2 h after glucose intake. The OGTT data were linked with the MBR data of the women who gave birth in 2009 using personal identification numbers. A total of 5150 women with OGTT results during pregnancy were found.

The laboratory in which the OGTT was performed was determined by the municipality of residence. To compare the MBR and HILMO GDM variables by using the OGTT laboratory data as a reference, we selected data from municipalities in which over 20% of pregnant women had OGTT laboratory data and more than five OGTT results were available. The sample of 11 438 women from 46 municipalities covering 19.2% of women who delivered in Finland in 2009 was used for the data validation detailed below (Figure 1). Women with multiple pregnancies (n = 369 women), the latter pregnancy of repeated pregnancies in 2009 (n = 9 women) and prepregnancy diabetes mellitus (DM) were excluded. The number of women with GDM and prepregnancy DM was identified from both the MBR 2009 and HILMO 1998–2009. Different register variables were checked against laboratory-verified OGTT results as a reference. The definitions for the GDM and DM cases are detailed in Table 1.

**Table 1.** Definitions for the cases of gestational diabetes mellitus and prepregnancy diabetes mellitus according to the laboratory-verified oral glucose tolerance test results used as a reference and the register variables to be tested.

|  | **Verified cases by abnormal OGTT (reference)** | **Register variables MBR 2009** | **Register variables HILMO 1998–2009** |
| --- | --- | --- | --- |
| **GDM case** | At least one abnormal laboratory-verified OGTT result during pregnancy* | Abnormal OGTT result  Insulin started  GDM ICD-10 code (O24.4 or O24.9) | GDM ICD-10 code (O24.4 or O24.9) during index pregnancy |
| **Non-GDM case** | Normal laboratory-verified OGTT result  No OGTT result in laboratory data | None of the above | None of the above |
| **Prepregnancy DM case** |  | DM ICD-10 code (E10–E11, E13 or O24.0–O24.3) | DM ICD-10 code (E10–E11, E13 or O24.0–O24.3) |

*According to the Finnish Current Care Guidelines.

OGTT, oral glucose tolerance test; MBR, Medical Birth Register; GDM, gestational diabetes mellitus; DM, diabetes mellitus.

**Results**

The findings on prepregnancy DM are detailed in Table 2. The proportion of women identified with prepregnancy DM was twice as high when using the variables from HILMO compared to when using the variables from MBR (1.5% vs 0.7%).

**Table 2.** Comparison of number and prevalence of prepregnancy diabetes mellitus according to the Medical Birth Register, HILMO or both the Medical Birth Register and HILMO in the validation sample (n =11 060).

| **Where prepregnancy DM was identified from** | **Prevalence of DM** | | **Normal laboratory-verified OGTT result**  **FALSE DM** | | **Abnormal laboratory-verified OGTT result**  **UNCERTAIN**  **DM** | | **No OGTT results in laboratory data**  **TRUE DM** | | **Total** |
| --- | --- | --- | --- | --- | --- | --- | --- | --- | --- |
|  | **n** | **%** | **n** | **%** | **n** | **%** | **n** | **%** | **%** |
| **MBR** | 82 | 0.7 | 0 | 0.0 | 9 | 11.0 | 73 | 89.0 | 100 |
| **HILMO** | 168 | 1.5 | 16 | 9.5 | 46 | 27.4 | 106 | 63.1 | 100 |
| ***HILMO, not MBR**** | *93* | *0.8* | *16* | *17.2* | *41* | *44.1* | *36* | *38.7* | *100* |
| **MBR or HILMO** | 175 | 1.6 | 16 | 9.1 | 50 | 28.6 | 109 | 62.3 | 100 |

DM, diabetes mellitus; OGTT, oral glucose tolerance test; MBR, Medical Birth Register; HILMO, Care Register for Health Care.

FALSE DM: probably misclassified cases because women with prepregnancy DM should not have normal OGTT results. UNCERTAIN DM: prepregnancy DM that could have been diagnosed with abnormal OGTT during pregnancy. TRUE DM: probably cases recorded correctly because women with prepregnancy DM should not undergo OGTT during pregnancy.

*Prepregnancy DM cases identified from HILMO but not from MBR.

The accuracy of the different variable combinations to identify GDM cases are shown in Table 3. The addition of GDM ICD-10 diagnosis codes to other MBR variables increased the accuracy of identifying GDM cases from 92.3% to 94.2% and the sensitivity from 43.8% to 71.5%, whereas the false-positive rate (FPR) increased from 1.6% to 3.0%. The addition of HILMO data slightly increased the accuracy to 94.8% and detection rate (DR) from 71.5% to 76.5%, whereas FPR did not change. The prevalence of GDM remained similar whether HILMO data were included or not. After the exclusion of prepregnancy DM according to the MBR, a total of 10,978 women were included in the validation sample (Figure 1).

**Table 3.** Accuracy to identify gestational diabetes mellitus cases (according to abnormal laboratory-verified OGTT results used as a reference) using different register data variables of 11,060 women in the validation sample.

|  |  | **Abnormal laboratory-verified OGTT result (reference)** | |  | **Accuracy†** | **Sens** | **FPR** | **PPV** | **NPV** | **Prevalence of**  **GDM** |
| --- | --- | --- | --- | --- | --- | --- | --- | --- | --- | --- |
| **Register-based criteria for GDM** |  | **Yes** | **No*** |  |  |  |  |  |  |  |
|  |  | **n** | **n** | **n** | **%** | **%** | **%** | **%** | **%** | **%** |
| 1) MBR: Abnormal OGTT result or insulin started | **Yes** | 536 | 157 | 693 | 92.3 | 43.8 | 1.6 | 77.3 | 93.3 | 6.3 |
|  | **No** | 687 | 9598 | 10 285 |  |  |  |  |  |  |
|  | **Total** | 1223 | 9755 | 10 978‡ |  |  |  |  |  |  |
| 2) Abnormal OGTT result or insulin started or GDM code in the MBR | **Yes** | 875 | 290 | 1165 | 94.2 | 71.5 | 3.0 | 75.1 | 96.5 | 10.5 |
|  | **No** | 348 | 9465 | 9813 |  |  |  |  |  |  |
|  | **Total** | 1223 | 9755 | 10 978‡ |  |  |  |  |  |  |
| 3) Abnormal OGTT result or insulin started or GDM code in the MBR or GDM code in HILMO | **Yes** | 904 | 292 | 1196 | 94.8 | 76.5 | 3.0 | 75.6 | 97.1 | 10.8 |
|  | **No** | 278 | 9411 | 9689 |  |  |  |  |  |  |
|  | **Total** | 1182 | 9703 | 10 885¶ |  |  |  |  |  |  |

Sens, sensitivity; FPR, false-positive rates; PPV, positive predictive value; NPV, negative predictive value; DM, diabetes mellitus; OGTT, oral glucose tolerance test; MBR, Medical Birth Register; GDM, gestational diabetes mellitus.

*Including women with normal laboratory-verified OGTT values and women without OGTT results in laboratory data.

†Proportion of correctly recorded variable combinations from registers.

Women with prepregnancy DM are excluded (‡n = 82 when using MBR and ¶ n = 175 when using MBR and HILMO).

OGTT was reported to have been performed in 3951 of 4673 (84.5%) laboratory-verified cases (normal or abnormal OGTT) and in 970 of 6305 (15.4%) cases without OGTT results in laboratory data (data not shown). When the GDM status was based only on the GDM ICD-10 code in the MBR (no ‘abnormal OGTT’ and no ‘insulin started’), 394 of 472 (83.5%) women were found to have the variable ‘OGTT performed’ in the MBR. Of these 394 women, 295 (74.9%) had unexpectedly abnormal laboratory-verified OGTT results.

These findings indicate that the usage of all three available MBR variables is superior to using only ‘abnormal OGTT’ and/or ‘insulin started’ variables. Furthermore, the accuracy to correctly identify recorded GDM cases was not improved when HILMO variables were added to the MBR variables. The ‘OGTT performed’ variable was not used for the data validation, but it was noticed to be recorded as ‘performed’ instead of ‘abnormal’ in 75% of women whose GDM statuses were based on ICD-10 diagnosis in the MBR. ‘OGTT performed’ was recorded in the MBR in 15.4% of women without OGTT results in laboratory data. This might have been a result of changing the municipality of residence between the period of OGTT sampling and the delivery. We did not have a reference value for prepregnancy DM. However, it is expected that women with prepregnancy DM do not participate in OGTT screening unless DM is identified as a result of abnormal OGTT during pregnancy. We noticed that a greater proportion of women with prepregnancy DM identified from MBR compared to HILMO did not have OGTT results in laboratory data (89.0% vs 63.1%) and none identified from MBR had normal laboratory-verified OGTT results. In addition, using the HILMO variables seemed to result in the overdiagnosis of prepregnancy DM, as its prevalence was twice as high compared to when using the MBR prepregnancy DM variables. This indicates the MBR is a more reliable source for identifying prepregnancy DM than the HILMO.

In conclusion, the identification of GDM from register data was decided to be based on MBR variables – including any of the following criteria indicating GDM: ‘abnormal OGTT’, ‘insulin started during pregnancy’ or ‘ICD-10 diagnosis code of GDM’.

**Figure 1.** Flow chart of 11 060 women with singleton pregnancies in the data validation sample. Women from 46 municipalities in which over 20% of women were screened using the oral glucose tolerance test and more than five OGTT results per municipality were available in six central laboratories in Finland (Oulu, Tampere, Kajaani, Pori, Lappeenranta and Seinäjoki) between 2008 and 2009 were chosen as the data validation sample.


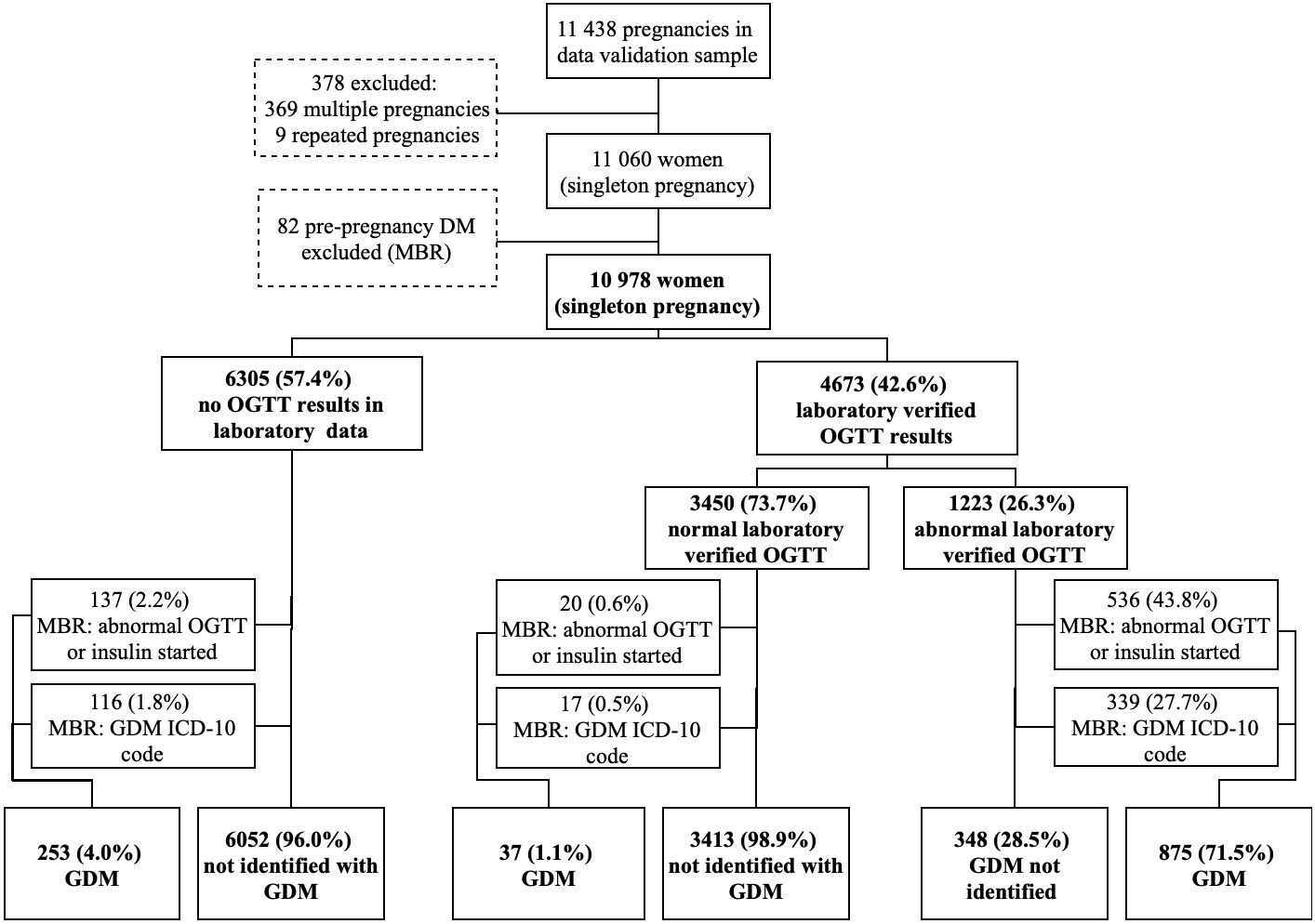


DM, diabetes; MBR, Medical Birth Register.
