# Supplementary material for: Cohort Profile: The Finnish Gestational Diabetes (FinnGeDi) Study
Source: Int J Epidemiol. 2020 May 6;49(3):762–763g. doi: 10.1093/ije/dyaa039 (PMC7394962; doi:10.1093/ije/dyaa039)
Supplement: dyaa039_Supplementary_Data [file dyaa039_supplementary_data.zip › dyaa039-Suppl_Data/ije-2019-08-1156-File011.docx]

**Supplement Table 1.** **Maternal glucose metabolism.**

Characteristics of glucose metabolism in women with gestational diabetes mellitus (GDM) and controls.

| **Characteristic** | **GDM**  **n** **= 1146** | **Controls**  **n = 1066** | **P-value*** |
| --- | --- | --- | --- |
| OGTT during pregnancy, n (%) | 1130 (98.6%) | 669 (62.8%) | < 0.001 |
| OGTT before 16 weeks of gestation, n (%) | 395 (34.5%) | 66 (6.2%) | < 0.001 |
| OGTT values, plasma glucose, mmol/l |  |  |  |
| Fasting | 5.3 ± 0.6 (1127) | 4.7 ± 0.3 (669) | < 0.001 |
| 1 h | 9.5 ± 1.9 (1118) | 7.1 ± 1.4 (667) | < 0.001 |
| 2 h | 7.4 ± 1.7 (1119) | 5.8 ± 1.1 (667) | < 0.001 |
| Number of abnormal values in OGTT, n (%) | (1,121) |  |  |
| 0 | 9 (0.8%)^a^ |  |  |
| 1 | 640 (57.1%) |  |  |
| 2 | 361 (32.2%) |  |  |
| 3 | 111 (9.9%) |  |  |
| Length of gestation at time of GDM diagnosed, n (%) |  |  |  |
| ≤ 20 gestational weeks | 327 (30.1%) (1085) |  |  |
| > 20 gestational weeks | 758 (69.9%) (1085) |  |  |
| Insulin or metformin treated GDM, n (%) | 221 (18.1%) (1123) |  |  |

GDM, gestational diabetes mellitus; OGTT, oral glucose tolerance test.

*Unadjusted based on student’s t test or χ^2^ test.

Data are presented as mean ± SD or number (percentages).

()Number of available information unless from all.

^a^Diagnosed by self-monitoring after normal OGTT result.
