# Supplementary material for: Cohort Profile: The Finnish Gestational Diabetes (FinnGeDi) Study
Source: Int J Epidemiol. 2020 May 6;49(3):762–763g. doi: 10.1093/ije/dyaa039 (PMC7394962; doi:10.1093/ije/dyaa039)
Supplement: dyaa039_Supplementary_Data [file dyaa039_supplementary_data.zip › dyaa039-Suppl_Data/ije-2019-08-1156-File012.docx]

**Supplement Table 2A-C.** **Clinical data of women in register-based arm.**

Comparison of baseline clinical characteristics and the Medical Birth Register variables of 58 587 women with singleton pregnancy in the register-based arm.

1. Comparison of all 58 587 women according to their GDM status.

| **Characteristic** | **GDM** | | **Controls** | |  |
| --- | --- | --- | --- | --- | --- |
|  | **All**  **n = 6583** | | **All**  **n = 52 004** | |  |
|  | **mean** | **SD** | **mean** | **SD** | **P-value^a^** |
| Age at delivery, years | 31.1 | 5.6 | 29.3 | 5.3 | < 0.001 |
| BMI prepregnancy, kg/m^2^ | 28.4 | 6.0 | 23.7 | 4.3 | < 0.001 |
| Parity, n | 1.25 | 1.6 | 1.02 | 1.4 | < 0.001 |
|  | **n** | **%** | **n** | **%** | **P-value^b^** |
| Primiparity | 2390 | 36.3 | 22 372 | 43.0 | < 0.001 |
| OGTT performed | 5812 | 88.3 | 19 227 | 37.0 |  |
| OGTT abnormal | 5155 | 78.3 |  |  |  |
| Insulin started | 972 | 14.8 |  |  |  |
| ICD-10 (O24.9 or O24.4) | 4671 | 71.0 |  |  |  |

1. Comparison of controls (n = 52 004) divided as groups according to OGTT verification.

| **Characteristic** | **Controls** | | | |  |
| --- | --- | --- | --- | --- | --- |
|  | **OGTT result verified**  **n = 19 227** | | **OGTT result not verified**  **n = 32 777** | |  |
|  | **mean** | **SD** | **mean** | **SD** | **P-value^a^** |
| Age at delivery, years | 30 | 5.3 | 29.0 | 5.2 | < 0.001 |
| BMI prepregnancy, kg/m^2^ | 26.1 | 4.9 | 22.3 | 3.1 | < 0.001 |
| Parity, n | 0.96 | 1.4 | 1.05 | 1.4 | < 0.001 |
|  | **n** | **%** | **n** | **%** | **P-value^b^** |
| Primiparity, n | 8914 | 46.4 | 13 458 | 41.1 | < 0.001 |

1. Comparison of women with GDM (n = 6 583) according to their entry MBR variable.

| **Characteristic** | **GDM** | | | |  |
| --- | --- | --- | --- | --- | --- |
|  | **OGTT abnormal or insulin started**  **n = 5413** | | **Only GDM**  **ICD-10 code**  **n = 1170** | |  |
|  | **mean** | **SD** | **mean** | **SD** | **P-value^a^** |
| Age at delivery, years | 31.2 | 5.6 | 31.1 | 5.7 | 0.627 |
| BMI prepregnancy, kg/m^2^ | 28.4 | 6.0 | 28.6 | 6.2 | 0.269 |
| Parity, n | 1.2 | 1.5 | 1.48 | 1.9 | < 0.001 |
|  | **n** | **%** | **n** | **%** | **P-value^b^** |
| Primiparity | 2037 | 37.6 | 353 | 30.2 | < 0.001 |
| OGTT performed | 5048 | 93.3 | 764 | 65.3 |  |
| OGTT abnormal | 5155 | 95.2 | − | − |  |
| Insulin started | 972 | 18.0 | − | − |  |
| ICD-10 (O24.9 or O24.4) | 3501 | 64.7 | 1 170 | 100 |  |

GDM, gestational diabetes mellitus; BMI, body mass index; OGTT, oral glucose tolerance test. P-value based on ^a^Student’s t-test or ^b^ χ^2^ test.
