# Supplementary material for: Cohort Profile: The Finnish Gestational Diabetes (FinnGeDi) Study
Source: Int J Epidemiol. 2020 May 6;49(3):762–763g. doi: 10.1093/ije/dyaa039 (PMC7394962; doi:10.1093/ije/dyaa039)
Supplement: dyaa039_Supplementary_Data [file dyaa039_supplementary_data.zip › dyaa039-Suppl_Data/ije-2019-08-1156-File013.docx]

**Supplement Table 3.** **Controls in case-control arm.**

Characteristics of 394 control women without oral glucose tolerance test result(s) during pregnancy in the case-control arm (total 1066 women).

| **Characteristic** | **Primiparas** | **Multiparas** |
| --- | --- | --- |
| Controls without indication for OGTT (very low risk) (total n = 319),n (% of all controls)^a^ | 238 (35.2%) | 81 (20.8%) |
| Controls without OGTT and risk factors (total n= 75),  n (% of all controls)^b^ | 63 (9.3%) | 12 (3.1%) |
| Age at delivery ≥ 25 years for primiparas or ≥ 40 years for multiparas, n (%)  (Range, years) | 51  (25.0–38.6) | 1  (40.8) |
| BMI ≥ 25 kg/m^2^, n (%)  (Range, kg/m^2^) | 2  (25.7–30.5) | 8  (25.7–34.9) |
| Family history of T2D, n (%) | 2 | – |
| History of macrosomic newborn ≥ 4500 g, n (%) | – | 2 |
| Two indications for OGTT, n (%) | 8 | 1 |

OGTT, oral glucose tolerance test; BMI, body-mass-index; T2D, type 2 diabetes.

^a^Very low risk controls included < 25-year-old primiparous women with BMI < 25 kg/m^2^ and without family history of diabetes and < 40-year-old multiparous women with BMI < 25 kg/m^2^ and without history of GDM or macrosomic newborn (birth weight > 4500 g).

^b^OGTT was indicated but not performed.
