# Supplementary material for: Cohort Profile: The Finnish Gestational Diabetes (FinnGeDi) Study
Source: Int J Epidemiol. 2020 May 6;49(3):762–763g. doi: 10.1093/ije/dyaa039 (PMC7394962; doi:10.1093/ije/dyaa039)
Supplement: dyaa039_Supplementary_Data [file dyaa039_supplementary_data.zip › dyaa039-Suppl_Data/ije-2019-08-1156-File014.docx]

**Supplement Figure 1.** **Subcohort of 5150 women.**

Flow chart of 5150 women with laboratory-verified oral glucose tolerance test results collected from the databases of six central laboratories serving two tertiary and four secondary level delivery hospitals (in Oulu, Tampere, Kajaani, Pori, Lappeenranta and Seinäjoki) between April 2008 and December 2009.


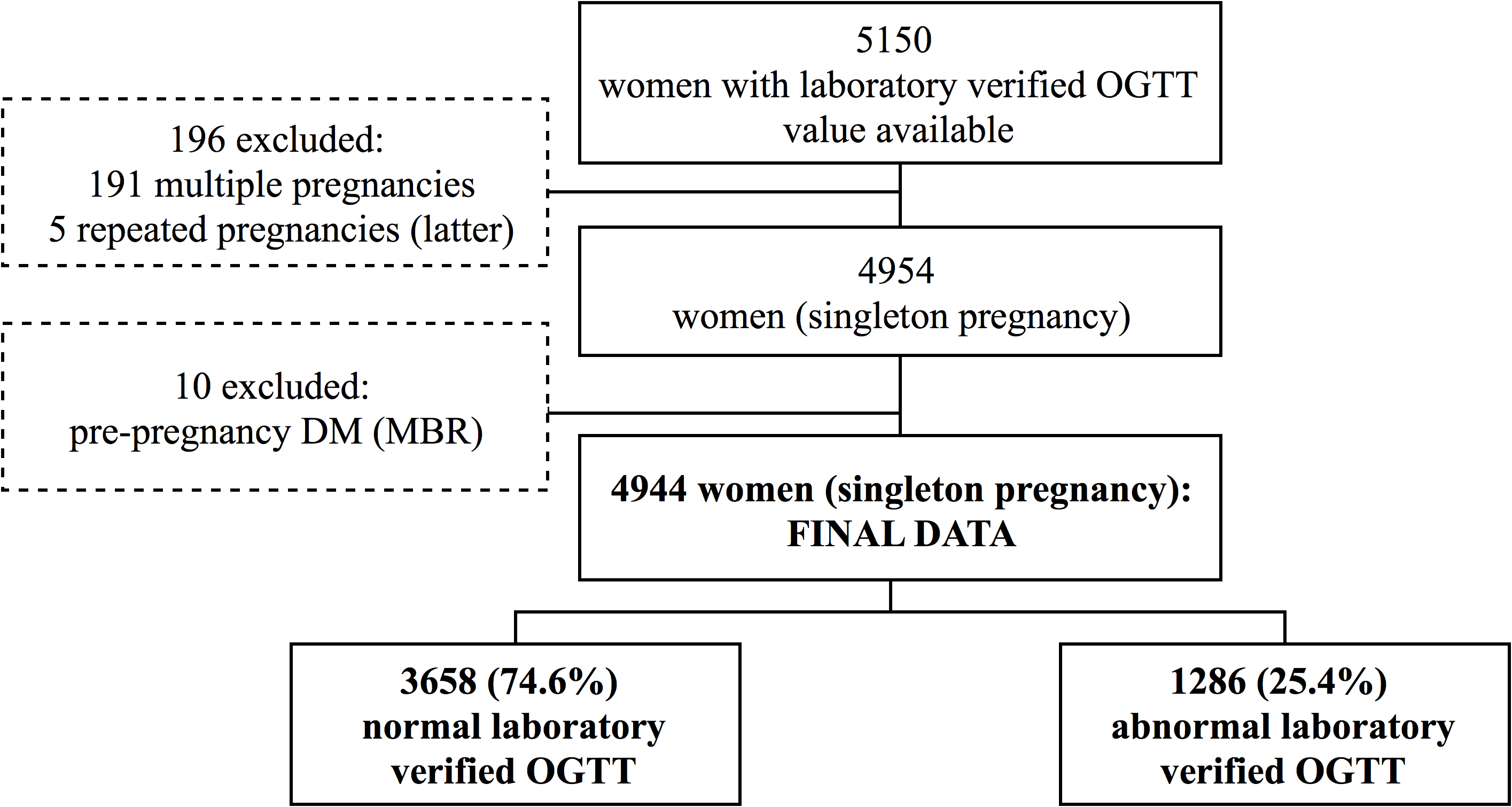


DM, diabetes; MBR, Medical Birth Register; OGTT, oral glucose tolerance test.
